# Differences in Gut Microbial Composition and Characteristics Among Three Populations of the Bamboo Pitviper (*Viridovipera stejnegeri*)

**DOI:** 10.1002/ece3.70742

**Published:** 2024-12-17

**Authors:** Jiaqi Zhang, Songwen Tan, Bing Lyu, Min Yu, Yue Lan, Ruixiang Tang, Zhenxin Fan, Peng Guo, Lei Shi

**Affiliations:** ^1^ Xinjiang Key Laboratory for Ecological Adaptation and Evolution of Extreme Environment Biology, College of Life Sciences Xinjiang Agricultural University Urumqi China; ^2^ Faculty of Agriculture, Forestry and Food Engineering Yibin University Yibin China; ^3^ Key Laboratory of Bioresources and Ecoenvironment (Ministry of Education), College of Life Sciences Sichuan University Chengdu China

**Keywords:** carbohydrate‐active enzymes, gut microbiota, metagenomic next‐generation sequencing, microbial diversity, *Viridovipera stejnegeri*

## Abstract

The gut microbiota contributes to host health by facilitating nutrient uptake, digestion, energy metabolism, intestinal development, vitamin synthesis, and immunomodulation, and plays an important role in the growth and reproduction of the animal itself. Considering the paucity of research on the gut microbiota of wild snakes, this study focused on bamboo pitviper (*Viridovipera stejnegeri*) populations from Anhui, Guizhou, and Hunan, with multiple fecal samples collected from each population (six, five, and three, respectively). Total microbial DNA was extracted from the fecal samples using metagenomic next‐generation sequencing and differences in gut microbial composition, abundance, and carbohydrate‐active enzymes (CAZymes) were analyzed and compared among the three populations. Results showed no significant variance in the α‐diversity of the gut microbes across the three populations, while principal coordinate analysis revealed significant differences in gut microbe composition. The four most abundant phyla in the gut microbiota of *V. stejnegeri* were Pseudomonadota, Bacteroidota, Actinomycetota, and Bacillota, while the four most abundant genera were *Salmonella*, *Citrobacter*, *Bacteroides*, and *Yokenella*. Linear discriminant analysis effect size demonstrated notable differences in gut microbial abundance among the three populations. Marked differences in CAZyme abundance were also observed across the microbial communities. Future studies should incorporate diverse ecological factors to evaluate their influence on the composition and function of gut microbiota.

## Graphical abstract

Considering the paucity of research on the gut microbiota of wild snakes, this study focused on bamboo pitviper (*Viridovipera stejnegeri*) populations from Anhui, Guizhou, and Hunan, with multiple fecal samples collected from each population (six, five, and three, respectively). Total microbial DNA was extracted from the fecal samples using metagenomic next‐generation sequencing and differences in gut microbial composition, abundance, and carbohydrate‐active enzymes (CAZymes) were analyzed among the three populations.

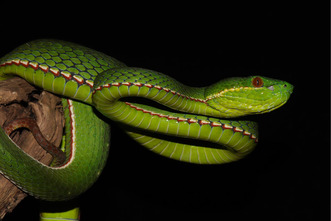

## 1 Introduction

The gut provides a rich and favorable ecological environment for microbial communities, facilitating a complex and dynamic equilibrium among microorganisms. This balance is subject to continuous modifications across different hosts or developmental stages, contributing to a variety of physiological (Hertli and Zimmermann 2022) and pathological processes, including metabolism (Lindsay, Metcalfe, and Llewellyn 2020; Wei et al. 2023), nutrient absorption (Flint et al. 2012), immune regulation (Siddiqui, Maciver, and Khan 2022; Wei et al. 2023), and host ecological behavior (O'Donnell et al. 2020). The composition and structure of normal gut microbiota serve as indicators for assessing animal health as well as diagnosing or preventing disease (Hu et al. 2017; Kundu, Blacher, and ElinavE 2017; Rosshart et al. 2017). Gut microbes are shaped by a multitude of factors, such as the evolutionary status (Kartzinel et al. 2019), feeding habits (Jiang et al. 2017), distribution (Qin et al. 2014), seasonal variation (Sun et al. 2016; Gao, Yang, and Shi 2023), and climate (Greenspan et al. 2020; Li, Liang, et al. 2020; Li, Yin, et al. 2020). However, the primary determinants affecting the composition of the gut microbiota are the genetic background and dietary habits of the host (Kovacs et al. 2011; Doré and Blottière 2015). Understanding these factors is crucial for studying the ecology and evolution of animal gut microbes. For example, significant sex‐based differences in microbial community structure have been reported in wild oviparous lizard ( *Calotes versicolor* ), with the *Bacteroide* and *Ochrobactrum* genera found to be dominant in wild females and males, respectively (Zhang et al. 2022). Differences in fecal microbial abundance and gene functional types have also been noted between populations of *Ptyas dhumnades* (Li, Sun, and Xu 2021). Additionally, the relative abundance of *Shigella* species in fecal samples of *Elaphe carinata* , noted for its lack of intestinal diseases, is considerably higher than that of two congenerics, suggesting a potential link to intestinal health (Lu et al. 2019).

Research has also shown that conditional or potentially pathogenic bacteria are often present in the animal gut microbiota. For example, potential pathogenic bacteria such as *Citrobacter*, *Trichococcus*, and *Erysipelothrix* have been detected in the intestines of wild *Rhabdophis tigrinus* (Tang, Yang, et al. 2019; Tang, Zhu, et al. 2019) and *R. subminiatus* (Tang, Yang, et al. 2019; Tang, Zhu, et al. 2019). Significant differences have also been found in the composition of gut microbiota among populations of *Phrynocephalus vlangalii* living at different altitudes, with changes corresponding to the altitudinal gradient (Zhang, Li, et al. 2018; Zhang, Yohe, et al. 2018). Furthermore, based on semi‐natural experiments, Bestion et al. (2017) demonstrated that increasing temperature can lead to a decrease in microbial diversity in the common lizard (*Zootoca vivipara*), potentially negatively impacting host survival. Conversely, gut microbiota can be regulated to enhance immune capacity in lizards, thus facilitating adaptation to climate change (Yang et al. 2024). Overall, these various factors can affect the composition and characteristics of gut microbiota.

In the current analyses of metabolic functions in vertebrates, carbohydrate metabolism in general appears as a major metabolic function (Zhou, Xu, and Zhou 2019; Liu et al. 2021; Jiang et al. 2023; Gao, Yang, and Shi 2023). The activity and stability of its carbohydrase enzymes contribute to the degradation of complex substrates, improves nutrient uptake by the animal, and participate in important physiological and pathological processes in various systems. Carbohydrate‐active enzymes (CAZymes) play a key role in carbon source metabolism. The CAZy database categorizes enzyme families that catalyze the degradation, modification, and biosynthesis of carbohydrates into five major classes and one related module. For example, glycoside hydrolases (GHs) are involved in the hydrolysis and rearrangement of glycosidic bonds, glycosyl transferases (GTs) participate in the formation of glycosidic bonds, polysaccharide lyases (PLs) function in the non‐hydrolytic cleavage of glycosidic bonds, carbohydrate esterases (CEs) are involved in the hydrolysis of carbohydrate esters and auxiliary activities (AAs) is a redox enzymes that act in conjunction with CAZymes, and carbohydrate‐binding modules (CBMs) facilitate adhesion to carbohydrates (Lombard et al. 2014; Wardman et al. 2022).

The bamboo pitviper (*Viridovipera stejnegeri*) is a common venomous snake with wide distribution across China and Vietnam (David 2002; Guo et al. 2022). It typically inhabits the rocks of streams, grassy and bushy areas, roadsides, vegetable fields, and rock crevices in mountainous areas at elevations ranging from 150 to 2200 m. These snakes primarily feed on mice, frogs, lizards, and birds (Zhao et al. 1998; Guo et al. 2022). Molecular phylogeny based on mitochondrial gene fragments and nuclear genes revealed significant genetic divergence between populations within this species (Guo et al. 2016). The Guizhou and Hunan populations showed closer relationship than that between them and Anhui population in the phylogenetic tree. Snakes, lacking the ability to chew, possess a robust intestinal digestion capacity and remarkably strong hunger tolerance. In the current study, we analyzed the composition, abundance, and CAZymes of gut microbes in different populations of *V. stejnegeri* using metagenomic next‐generation sequencing. Our main goal was to compare the gut microbiota in the three different populations and investigate the potential relationship between habitat factors and gut microbes as well as the crucial roles played by gut microbes during carbohydrate metabolism within populations of *V. stejnegeri*.

## 2 Materials and Methods

### 2.1 Collection Sites

**FIGURE 1:**
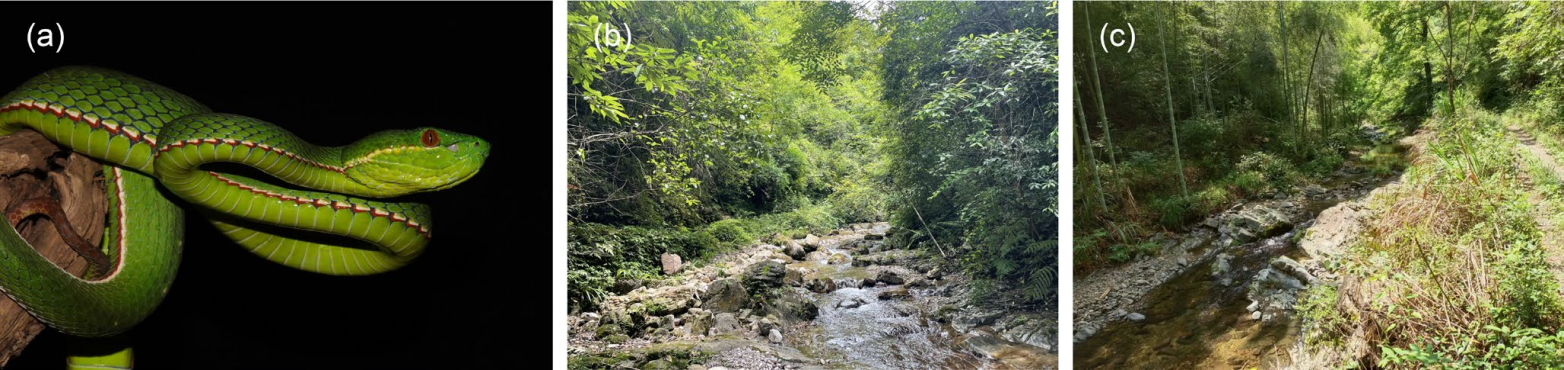
*Viridovipera stejnegeri* (a) and its habitat in Guizhou (b) and Anhui (c).

Samples were collected from three different localities during 2022–2023, including Huangshan City, Anhui Province (118°0′–118°2′  E and 30°14′ N, 197–220 m), Guiyang City, Guizhou Province (106°9′–106°49′ E and 26°77′–27°11′ N, 941–1232 m), and Yongxing County, Hunan Province (112°22′–112°23′  E, 26°14′–26°15′ N, 318–319 m) in China. The three locations feature subtropical monsoon climates. The habitats of the Anhui and Hunan populations are primarily composed of scrub and bamboo forests adjacent to streams, while the habitats of the Guizhou population predominantly consists of wooded areas adjacent to streams (Figure 1).

### 2.2 Sample Collection and DNA Extraction

In total, 14 snake samples were collected, including six (three males and three females) from Huangshan, five (three males and two females) from Guiyang City, and three (two males and one female) from Yongxing County. All individuals were healthy adults. Upon capture, the snakes were immediately placed in sterilized plastic collection boxes overnight to collect feces using sterile disposable gloves. The feces were collected in 2‐mL collection tubes containing 50% glycerin (Fuyu Fine Chemical Co. Ltd, Tianjin, China) and placed in liquid nitrogen for rapid freezing, with subsequent preservation in −80°C refrigerator at the Yibin Key Laboratory of Zoological Diversity and Ecological Conservation, Yibin University (YBU). The snakes were released at the collection sites after obtaining fecal samples. Genomic DNA was extracted from the gut contents by the commercial laboratory, Novogene (Beijing, China) utilizing a rapid plus DNA lib prep kit for illumina (RK20208) (Shanghai Molecular Research and Development Center, Shanghai, China) following the manufacture instruction. Sample collection permission (YBU2020007) was issued by the Animal Care and Ethics Committee of Yibin University.

### 2.3 Library Construction, Quality Control (QC), and Sequencing

Approximately 0.2 μg of DNA per sample was used as input material for the DNA library preparations. In brief, the genomic DNA samples were fragmented by sonication to a size of 350 bp, then polished, A‐tailed, and ligated with the full‐length adapter for Illumina sequencing, followed by polymerase chain reaction (PCR) amplification. The resulting PCR products were purified using the AMPure XP system (Beverly, USA). Subsequently, library quality was assessed using the Agilent 5400 system (Agilent, USA) and quantified by quantitative PCR (QPCR) (1.5 nM). The qualified libraries, each with an effective concentration over 2 nM, were pooled and sequenced on the Illumina HiSeq 2500/MiSeq platform using the PE150 strategy by Novogene Bioinformatics Technology Co. Ltd., (Beijing, China). The original fluorescence image files obtained were transformed to short reads (raw reads) by base calling and BclToFastq, and recorded in FASTQ format (Cock et al. 2010).

### 2.4 Data Analyses

The quality of the raw reads was assessed using Fastp v0.23.1 (Chen et al. 2018), with read pairs discarded if: (1) adapter contamination was present in either read; (2) more than 10% of bases were uncertain in either read; and (3) the proportion of low‐quality (Phred quality < 5) bases exceeded 50% in either read.

Due to the unavailability of the whole‐genome sequence for *V. stejnegeri*, the available genome sequence of *Protobothrops mucrosquamatus* , which is a close relative of *V. stejnegeri* (Figueroa et al. 2016; Li, Liang, et al. 2020; Li, Yin, et al. 2020), was used as a host reference genome, downloaded from the NCBI database (https://ftp.ncbi.nlm.nih.gov/genomes/al/GCA/001/527/595/GCA001527695.3_P.Mucros_1.0/). Sequence alignment was performed using Bowtie2 v2.4.1 (Langmead and Salzberg 2012; Langmead et al. 2019) to remove the host sequence. After host removal, the sequences were aligned to standard archaea, bacteria, human, UniVec_Core, and viral databases using Kraken2 v2.0.7 to obtain annotation information and abundance tables (Wood, Lu, and Langmead 2019). Target sequences were then partitioned into smaller k‐mer segments for assembly using Megahit v1.2.9 (Li et al. 2015, 2016) with the parameters (‐t 8 ‐m 0.95 ‐‐min‐contig‐len 300 ‐‐k‐min 51 ‐‐k‐max 127 ‐‐k‐step 20). The assembled sequences were clustered using Cd‐hit v4.8.1 (Fu et al. 2012) to obtain de‐redundant sequences with the parameters (−c 0.95 ‐aS 0.9 ‐g 1 ‐sc 1 ‐sf 1 ‐T 8 ‐M 8000). Protein sequences were predicted using Prodigal v2.6.3 (Hyatt et al. 2010). Finally, the annotated protein sequences were obtained by comparison against the carbohydrate database v3.0.5 with Run_dbcan (Zhang, Li, et al. 2018; Zhang, Yohe, et al. 2018), with quantification performed using Salmon v0.14.1 (Patro et al. 2017).

Grouped percentage stacked column charts representing species abundance were produced using the Wekemo Bioincloud platform (https://bioincloud.tech/task‐meta). Several diversity indices were calculated in R v4.3.1 (R Development Core Team 2020), including α‐diversity indices such as abundance‐based coverage estimation, Simpson's diversity index, Chao1 estimator, Shannon‐Weiner index, observed species, and Goods's coverage index. The Kruskal–Wallis test was completed to compare α‐diversity indices among the three populations.

Principal coordinate analysis (PCoA) using Bray‐Curtis distance was performed to detect differences in the composition of gut microbiota across the three populations of *V. stejnegeri*. Adonis analysis of variance (ANOVA) was employed to assess significant differences among the three populations using R v4.3.1. Additionally, linear discriminant analysis (LDA) effect size (LEfSe) was used to examine significant disparities in the abundance of gut bacteria among the three populations at the phylum to genus levels and identify components with notable differences. LDA was then applied to assess the impact of each component on differences in abundance (Segata et al. 2011), with the results visualized using R v4.3.1. The linear discriminant analysis criterion was set to a log‐transformed value greater than or equal to 2 with a base of 10.

## 3 Results

Detail characteristics of sequences and data were provided in the Appendix S1.

### 3.1 Composition and Characteristics of Gut Microbiota in *V. Stejnegeri*

**FIGURE 2:**
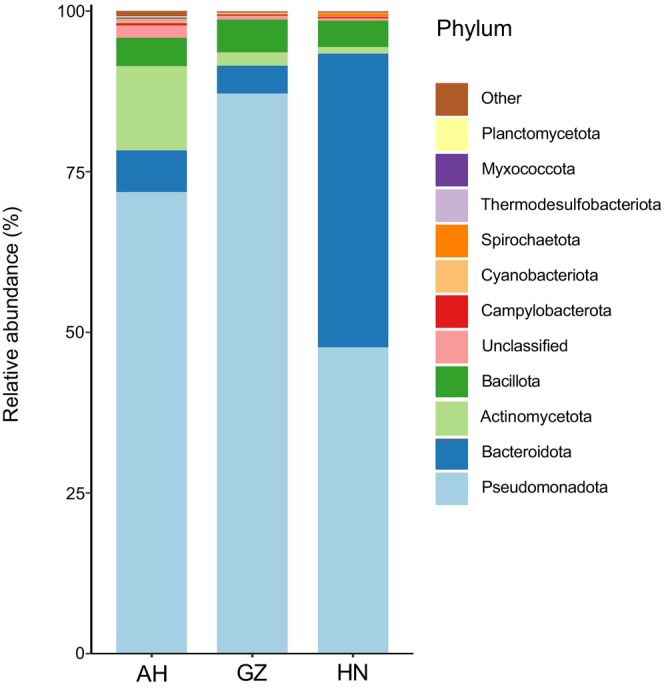
Relative abundance of gut bacteria in three populations of *V. stejnegeri* at the phylum level. *y*‐axis represents the three sampling areas of Anhui (AH), Guizhou (GZ) and Hunan (HN), different colors represent classification of bacteria at the phylum level.

**FIGURE 3:**
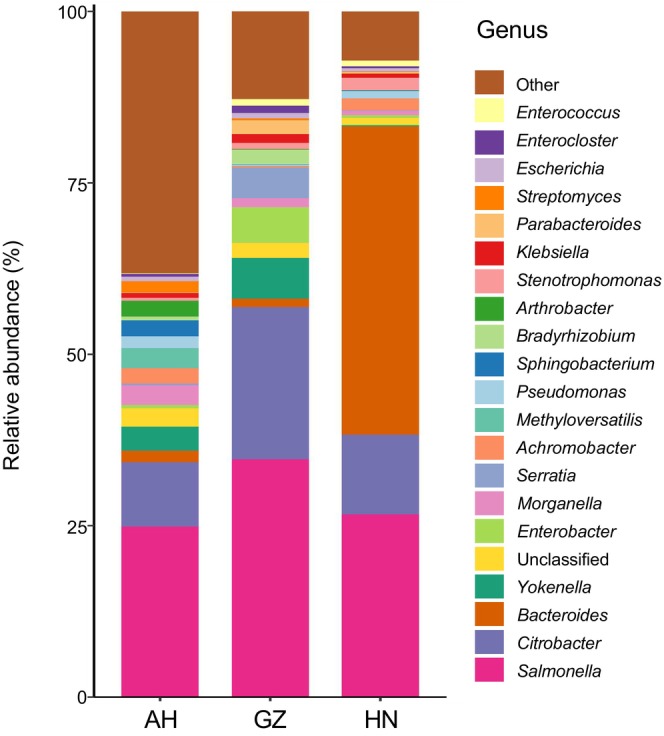
Relative abundance of gut bacteria in three populations of *V. stejnegeri* at the genus level. *y*‐axis represents the three sampling areas of Anhui (AH), Guizhou (GZ) and Hunan (HN), different colors represent classification of bacteria at the genus level.

Results indicated that the gut microbiota of *V. stejnegeri* predominantly consisted of Bacteria (82.19%), Eukaryota (17.44%), Viruses (0.22%), and Archaea (0.15%). A total of 9435 bacterial species, belonging to 47 phyla, 101 classes, 214 orders, 514 families, and 1951 genera, were detected. At the bacterial phylum level, the four most abundant groups, with a relative abundance ≥ 3%, were Pseudomonadota (72.12% ± 20.72%), Bacteroidota (14.11% ± 19.00%), Actinomycetota (6.58% ± 8.74%), and Bacillota (4.58% ± 2.79%) (Figure 2). The dominant phyla in the three different populations are listed in Table 1. At the genus level, the four most abundant genera, with a relative abundance ≥ 3%, were *Salmonella* (28.76% ± 23.53%), *Citrobacter* (14.36% ± 13.56%), *Bacteroides* (10.78% ± 20.23%), and *Yokenella* (3.65% ± 6.08%) (Figure 3). Variations in dominant genera were observed across the different populations (Table 2).

**TABLE 1:** Dominant phyla in three populations of *Viridovipera stejnegeri* (relative abundance ≥ 3%).

Population	Phylum			
	Pseudomonadota (%)	Actinomycetota (%)	Bacteroidota (%)	Bacillota (%)
Anhui	71.83 ± 17.46	13.11 ± 10.71	6.46 ± 5.15	4.41 ± 3.30
Guizhou	87.16 ± 6.52	2.10 ± 1.18	4.31 ± 4.56	5.09 ± 2.99
Hunan	47.65 ± 22.15	1.00 ± 0.32	45.73 ± 20.96	4.09 ± 1.17

**TABLE 2:** Dominant genera in three populations of *Viridovipera stejnegeri* (relative abundance ≥ 3%).

Population	Genus					
	*Salmonella* (%)	*Citrobacter* (%)	*Bacteroides* (%)	*Yokenella* (%)	*Enterobacter* (%)	*Serratia* (%)
Anhui	24.88 ± 24.82	9.38 ± 12.47	—	3.47 ± 6.82	—	—
Guizhou	34.70 ± 19.62	22.19 ± 14.08	—	5.94 ± 7.82	5.21 ± 9.46	4.46 ± 8.60
Hunan	26.65 ± 20.03	11.62 ± 7.59	44.92 ± 20.46	—	—	—

### 3.2 Population Differences in Gut Microbiota of *V. Stejnegeri*

**FIGURE 4:**
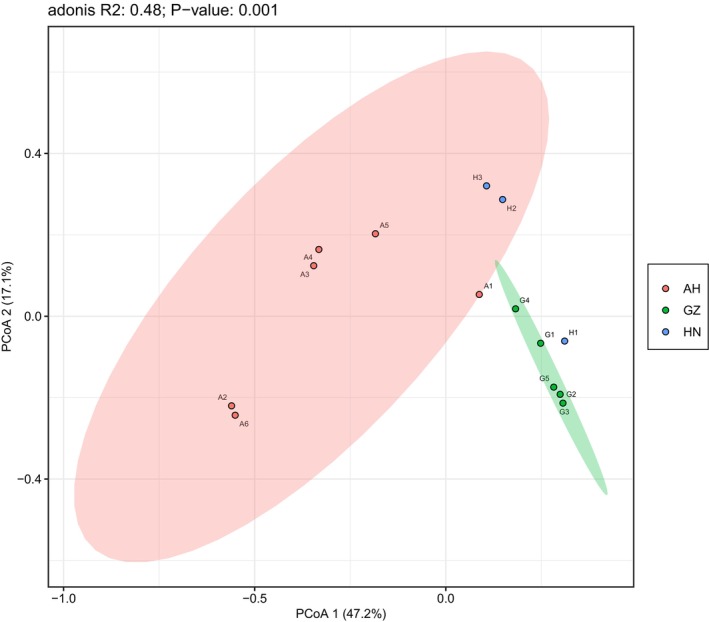
PCoA of Bray‐Curtis distance matrix for bacterial diversity differences among three populations of *V. stejnegeri*. A1, A2, A3, A4, and A5 are five independent samples from the Anhui (AH) province, G1, G2, G3, and G4 from the Guizhou (GZ), and H1, H2 and H3 from the Hunan (HN), respectively.

**FIGURE 5:**
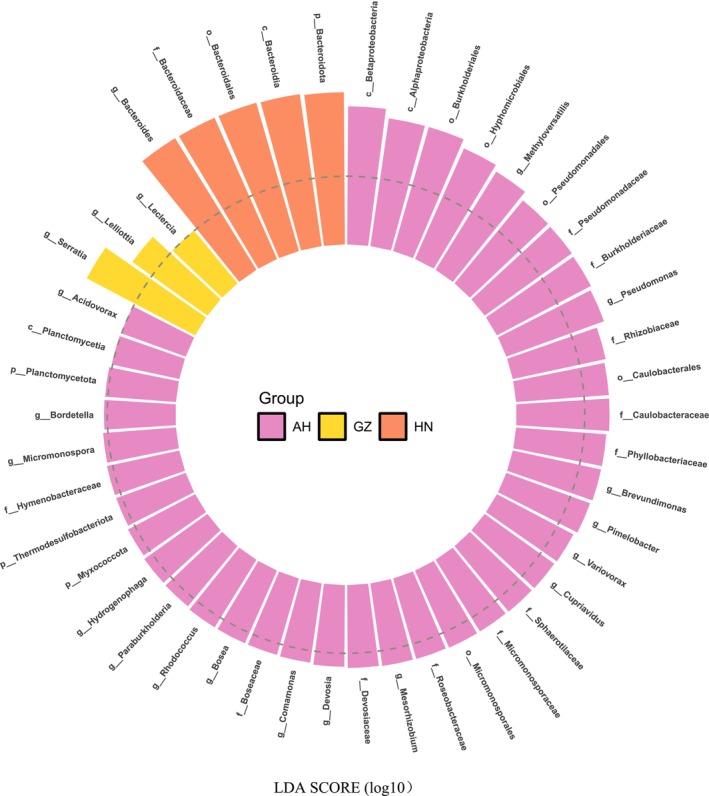
LEfSe of the three populations of *Viridovipera stejnegeri*. LDA scores identify significantly different taxa among Anhui (AH), Guizhou (GZ), and Hunan (HN) populations. Degree of influence of a taxon is expressed by bar length. Only taxa meeting a significant LDA threshold > 2 are shown. Letters p, c, o, f, and g represent phylum, class, order, family, and genus, respectively.

The α‐diversity analyses revealed no significant differences in the intestinal flora among the three populations of *V. stejnegeri* (Table 3). The PCoA results indicated that 64.3% of the variance was explained by PCoA 1 and PCoA 2, with significant differences in gut microbial diversity among the three populations (Figure 4, *R* ^2^ = 0.48, *p* = 0.01). PCoA results also indicated that all samples from Anhui clustered with the H2 and H3 samples from Hunan, while all samples from Guizhou clustered with the H1 sample from Hunan. Based on LEfSe, gut microbial abundance among the three populations differed significantly, with 38 microbial taxa from Anhui, three from Guizhou, and five from Hunan explaining the differences among the three populations (Figure 5). Notably, the Anhui population was characterized by taxa in the phyla Pseudomonadota, Actinomycetota, Myxococcota, Thermodesulfobacteriota, Bacteroidota, and Planctomycetota. In contrast, the Guizhou population was characterized by taxa in the phylum Pseudomonadota, and the Hunan population by taxa in the phylum Bacteroidota (Figure 5).

**TABLE 3:** Gut microbial α‐diversity in *Viridovipera stejnegeri* across three populations.

Population	Alpha diversity index					
	Abundance‐based coverage estimation, ACE	Simpson's diversity index	Chao1 estimator	Shannon‐weiner index	Observed species	Goods's coverage index
Anhui	10257.73 ± 1375.21	0.93 ± 0.03	10296.94 ± 1486.57	4.02 ± 0.82	9351.33 ± 2330.35	0.9996 ± 0.0002
Guizhou	9166.42 ± 1592.00	0.91 ± 0.01	9319.98 ± 1322.57	3.13 ± 0.33	7731.8 ± 1718.51	0.9994 ± 0.0001
Hunan	9208.47 ± 468.36	0.92 ± 0.01	9120.22 ± 174.62	3.15 ± 0.07	6891.33 ± 152.53	0.9992 ± 0.0001

### 3.3 Prediction of CAZymes in Gut Microbiota of *V. Stejnegeri*

**FIGURE 6:**
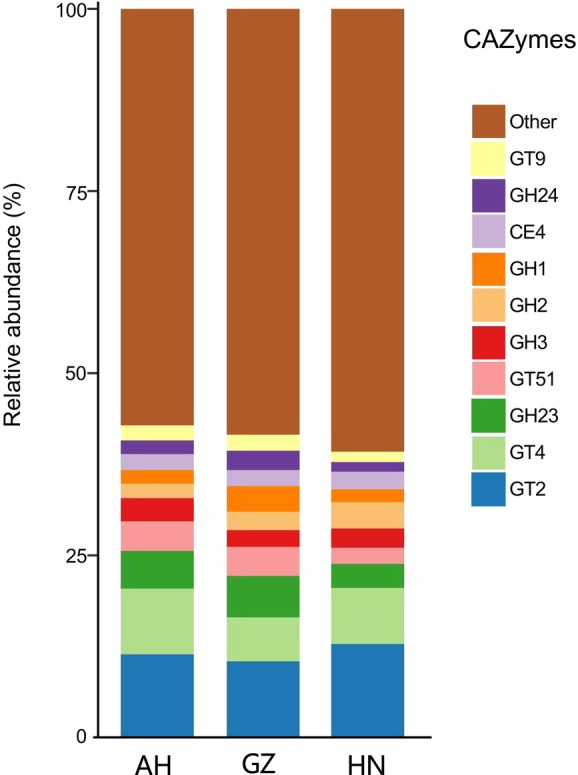
Relative abundance of CAZymes in *Viridovipera stejnegeri* across three populations. *y*‐axis represents the three sampling areas of Anhui (AH), Guizhou (GZ) and Hunan (HN), different colors represent classification of CAZymes.

**FIGURE 7:**
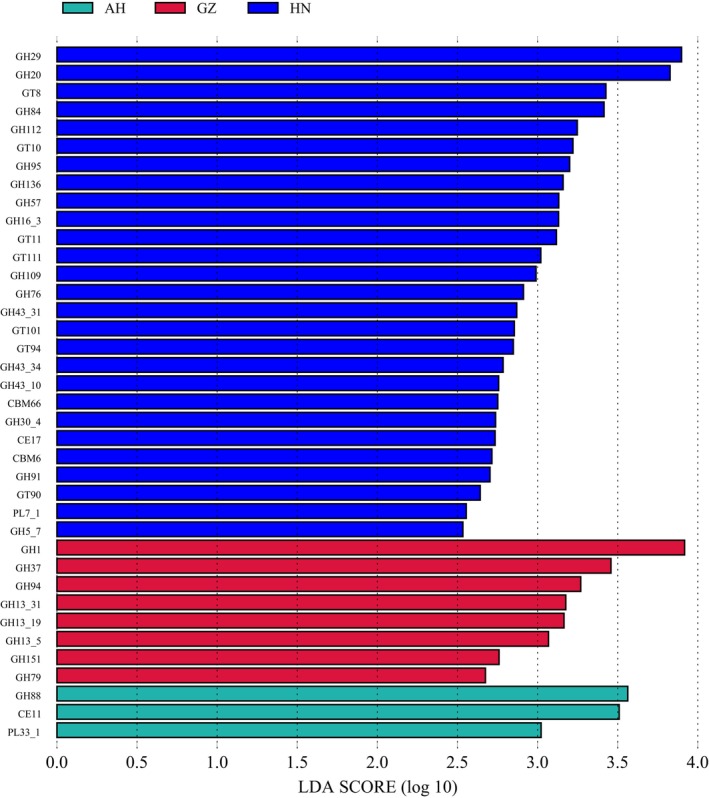
Differences in CAZymes among three populations of *Viridovipera stejnegeri*. LDA scores reflect differences in relative abundance of CAZymes among the three populations in Anhui (AH), Guizhou (GZ) and Hunan (HN).

The top four CAZymes in the gut microbiota of *V. stejnegeri* were GT2 (9.28% ± 1.57%), GT4 (6.16% ± 1.79%), GH23 (4.06% ± 1.30%), and GT51 (3.02% ± 1.00%) (relative abundance ≥ 3%) (Figure 6). No significant difference in α‐diversity was detected in the gut microbiota among the three populations (Kruskal–Wallis test, *p* > 0.05). PCoA also showed no significant differences in the diversity of CAZymes among the three populations (Adonis *R* ^2^ = 0.18, *p* = 0.26). However, LEfSe showed that CAZyme abundance differed significantly among the three populations. Key CAZymes and their categories for each population are provided in Figure 7.

## 4 Discussion

In this study, we conducted the first metagenomic next‐generation sequencing analysis of the gut microbiome of the common Asian bamboo pitviper *V. stejnegeri*. Results identified Pseudomonadota, Bacteroidota, Actinomycetota, and Bacillota as the most dominant phyla, contrasting with previous studies on snakes that identified Proteobacteria, Bacteroidetes, and Firmicutes (see Appendix S2), demonstrating that dominant microbes differ among different snake species. Our findings indicated the main difference between *V. stejnegeri* and other snakes was a larger abundance of Actinobacteria in *V. stejnegeri*. In addition, compared to other snakes, our findings showed that *V. stejnegeri* possessed a unique gut microbiota, predominantly comprised of Actinobacteria. In contrast, lizards have been shown to harbor a gut microbiota primarily composed of Firmicutes, Bacteroidetes, and Proteobacteria (Hong et al. 2011; Kohl et al. 2017; Zhang, Li, et al. 2018; Zhang, Yohe, et al. 2018; Zhu et al. 2022; Baldo et al. 2023; Gao, Yang, and Shi 2023). Thus, while the dominant bacterial communities in snakes and lizards appear to be largely influenced by Proteobacteria and Bacteroidetes, their composition and abundance at the phylum level exhibit distinct differences.

Molecular phylogeny analyses indicated that the Guizhou and Hunan populations exhibited a closer genetic relationship within the three populations (Guo et al. 2016). Based on the PCoA results, however, the bacterial diversity among the three populations was not consistent with the phylogenetic relationships (Figure 4), suggesting that bacterial diversity is not influenced by population relatedness. Our findings demonstrated that the three populations shared similar dominant microbial phyla. At the genus level, the dominant genera with relative abundance greater than 3%, except for *Salmonella* and *Citrobacter*, distinct dominant genera were observed in different populations, including *Yokenella*, *Enterobacter*, and *Serratia* in the Guizhou population, *Bacteroides* in the Hunan population and *Yokenella* in the Anhui population.

Previous studies have showed that reptiles, such as iguanas, turtles, crocodiles, and snakes, can act as vectors for transmitting bacteria to humans, potentially causing paratyphoid fever (Cohen et al. 1980; Mermin, Hoar, and Angulo 1997; Waterman et al. 1990; Schröter et al. 2004; Grupka, Ramsay, and Bemis 2006; McLaughlin, Cochran, and Dowd 2015). In the current study, *Salmonella* was the most abundant genus in the gut microbiota of *V. stejnegeri*, with *S. enterica* , the most prevalent species within the genus (32.01% ± 23.73%), constituting 98.42% of the *Salmonella* detected. In addition, the conditionally pathogenic bacterium *Citrobacter*, a facultative anaerobe known to cause enteritis in animals (Mundy, Macdonald, and Dougan 2010), was also found at a relatively high abundance, second only to *Salmonella*. The high abundance of *Salmonella*, *Citrobacter*, and *S. enterica* in gut of *V. stejnegeri* suggests us to pay more attention to conservation and management in wild snakes.

The composition and abundance of the host gut microbiota are influenced by multiple factors (Zhu et al. 2022; Zhang, Li, et al. 2018; Zhang, Yohe, et al. 2018). Differences in gut microbes may contribute to the adaptation of different populations to different habitats, but the intrinsic mechanisms of how gut microbes are regulated to adapt to different environments need to be analyzed in more depth. The three geographic populations of *V. stejnegeri* were generally consistent in latitude, climate, and prey but varied in altitude and habitat. Notably, the Anhui, Hunan, and Guizhou populations are located at altitudes of 197–220 m, 318–319 m, and 941–1232 m, respectively. Furthermore, the Anhui and Hunan population habitats are mainly characterized by shrub and bamboo forests adjacent to streams, while the Guizhou population habitat primarily features wooded areas alongside streams. Here, PCoA revealed significant differences in the intestinal microflora among the three populations (Figure 4, *R* ^2^ = 0.48, *p* = 0.01), suggesting that altitude and habitat likely influence the composition and abundance of gut microbes.

Previous Kyoto Encyclopedia of Genes and Genomes (KEGG) analysis of *P. dhumnades* has shown that genes related to metabolism are most prominent at the first level, encompassing six types of biometabolic pathways, with carbohydrate metabolism being the most abundant at the second level, involving 43 pathways (Li, Sun, and Xu 2021). Metabolic profiling has also shown that carbohydrate metabolism is highly dominant in *Crotalus horridus* and is the main metabolic pathway in *R. subminiatus* (McLaughlin, Cochran, and Dowd 2015; Tang, Yang, et al. 2019; Tang, Zhu, et al. 2019). The CAZyme annotations for *V. stejnegeri* showed that three of the top four enzymes in abundance belong to the GTs family. Zhu et al. (2015) suggested that GTs play an important role in the adaptation and pathogenicity of human microorganisms. And a large number of pathogenic bacteria were found in the intestinal microorganisms of *V. stejnegeri*, so we speculate that GTs may also play an important role in the pathogenicity of the microorganisms of *V. stejnegeri*. The gut microbiota CAZyme annotations for *V. stejnegeri* showed that the Anhui and Guizhou populations were mainly composed of GT2 (8.93% ± 1.68%, 8.82% ± 1.29%), GT4 (6.97% ± 2.25%, 5.04% ± 0.90%), GH23 (4.14% ± 1.32%, 4.79% ± 0.98%), and GT51 (3.31% ± 0.74%, 3.35% ± 0.40%), while the Hunan population was primarily composed of GT2 (10.73% ± 0.57%), GT4 (6.39% ± 0.28%), and GH2 (3.05% ± 0.14%) (relative abundance ≥ 3%). These findings show that the composition and abundance of dominant CAZymes differed among the three populations. The LEfSe results further highlighted marked disparities in CAZyme abundance across the three populations. Additionally, certain Proteobacteria strains (Gammaproteobacteria, Enterobacterales, Enterobacteriaceae, *Salmonella*) and a higher proportion of certain Bacteroidetes strains (Bacteroidia, Bacteroidales, Bacteroidaceae, *Bacteroides*) (relative abundance ≥ 40%) were noted in the Hunan population. *Bacteroides* species, which metabolize polysaccharides and oligosaccharides, provide nutrition and vitamins to the host and other intestinal microbial residents (Zafar and Saier 2021). It was speculated that this difference ultimately led to differences in CAZymes composition and abundance among the three populations. However, the specific substrates and chemical mechanisms acted upon by these CAZymes in snake intestines remain unclear. Further research is needed to elucidate how the gut microbiota affects specific carbohydrate metabolism mechanisms.

## 5 Conclusions

The composition and abundance of gut microbiota in *V. stejnegeri* differed from those of other examined snake species. Notably, the gut bacteria of *V. stejnegeri* encompassed 47 phyla, 101 classes, 214 orders, 514 families, and 1951 genera. The top four most abundant phyla were Proteobacteria, Bacteroidetes, Actinobacteria, and Firmicutes, respectively, while the four most abundant genera were *Salmonella*, *Citrobacter*, *Bacteroides*, and *Yokenella*, with *S. enterica* , a pathogenic intestinal bacterium, showing the highest relative abundance. The four most abundant CAZymes in the gut microbiota of *V. stejnegeri* were GT2, GT4, GH23, and GT51. Significant differences were observed in the PCoA and LEfSe analyses of the gut microbiota among the three populations, as well as in the composition and abundance of CAZymes. Our study indicated that these population differences in gut microbial composition and abundance were not related to genetic differentiation but may be associated with differences in altitude and habitat. We hypothesized that variations in *Bacteroides* contributed to differences in CAZyme composition and abundance among the three populations. Future research should expand the host range to further explore the relationship between more geographic populations and gut microbiota, and to analyze the specific functions of the gut microbiota in conjunction with gut contents.

## Author Contributions

**Jiaqi Zhang:** conceptualization (lead), data curation (lead), formal analysis (lead), methodology (equal), resources (equal), software (equal), validation (equal), visualization (equal), writing – original draft (lead). **Songwen Tan:** conceptualization (equal), methodology (equal), resources (equal), supervision (equal), validation (equal). **Bing Lyu:** conceptualization (equal), formal analysis (equal), supervision (equal). **Min Yu:** conceptualization (equal), formal analysis (equal), methodology (equal), supervision (equal). **Yue Lan:** methodology (supporting), resources (supporting), software (supporting), visualization (supporting). **Ruixiang Tang:** methodology (equal), resources (supporting), software (supporting), validation (equal). **Zhenxin Fan:** conceptualization (equal), methodology (supporting), resources (supporting), software (supporting), supervision (equal), writing – review and editing (equal). **Lei Shi:** conceptualization (supporting), data curation (equal), formal analysis (equal), methodology (equal), supervision (lead), validation (equal), visualization (equal), writing – review and editing (lead). **Peng Guo:** conceptualization (lead), funding acquisition (supporting), methodology (supporting), project administration (supporting), supervision (equal), writing – review and editing (lead).

## Conflicts of Interest

The authors declare no conflicts of interest.

## Supporting information


**Appendix S1.** Characteristics of sequences by Metagenomic next‐generation.

**Appendix S2.** Dominant bacteria of gut microbiota in different snake species.

## Data Availability

The data supporting the findings of this study are publicly available from the National Center for Biotechnology Information (NCBI) Sequence Read Archive (SRA) at https://www.ncbi.nlm.nih.gov/bioproject/PRJNA1108923 and https://www.ncbi.nlm.nih.gov/bioproject/PRJNA1108898, reference number are PRJNA1108923 and PRJNA1108898.
