# Supplementary material for: Differences in Gut Microbial Composition and Characteristics Among Three Populations of the Bamboo Pitviper (Viridovipera stejnegeri)
Source: Ecol Evol. 2024 Dec 17;14(12):e70742. doi: 10.1002/ece3.70742 (PMC11651729; doi:10.1002/ece3.70742)
Supplement: Supplementary file 1 — Appendix S1. Characteristics of sequences by Metagenomic next‐generation. [file ECE3-14-e70742-s001.docx]

Appendix S1

**Metagenomic next-generation sequencing**

In total, 76.38 gigabytes (GB) of raw data, averaging 5.46 GB per sample, and 509 209 214 sequences (Anhui: 214 833 000; Guizhou: 179 543 666; Hunan: 114 832 548) were obtained from the 14 samples of *V. stejnegeri* across three sampling localities. After QC filtering, 75.16 GB of clean data, averaging 5.38 GB per sample, and 500 924 546 sequences (Anhui: 212 806 018; Guizhou: 178 779 316; Hunan: 109 399 212) were obtained. The base percentages of Q20 and Q30 exceeded 90%, and the GC content was above 40%, indicating high sequencing accuracy. The effective data from all samples exceeded 95%, indicating that most sequences could be annotated and sampled samples were sufficient and representative for subsequent analyses (Table 1). Sequence length, TPM (transcripts per kilobase of exon model per million mapped reads) values, and number of reads are detailed in Table 2.

Table 1 Quality of metagenomic next-generation sequencing data from three populations of *Viridovipera stejnegeri* (average values)

| Population | Q20 (%) | Q30 (%) | GC content (%) | Effective rate (%) |
| --- | --- | --- | --- | --- |
| Anhui (AH) | 97.56 | 93.50 | 48.55 | 99.06 |
| Guizhou (GZ) | 96.74 | 91.54 | 47.93 | 99.59 |
| Hunan (HN) | 97.54 | 93.59 | 43.55 | 95.54 |

Notes: Q20 and Q30: percentage of bases with Phred values > 20 and > 30, respectively, against total bases from Illumina HiSeq 2500/MiSeq; GC Content: percentage of G and C bases against total number of bases; Effective rate: ratio of valid data retained after filtering original data.

Table 2 Salmon quantitative analysis of gut microbiota samples from three populations of *Viridovipera stejnegeri* (average values)

| Population | Length (bp) | Effective Length (bp) | TPM | Num Reads |
| --- | --- | --- | --- | --- |
| Anhui | 439.22 | 225.69 | 9.94 | 86.29 |
| Guizhou | 530.26 | 277.60 | 10.26 | 101.29 |
| Hunan | 634.17 | 399.12 | 9.60 | 106.09 |

Notes: Length (bp): actual length of original FASTQ sequence; Effective length (bp): FASTQ sequence effective length; TPM value (transcripts per kilobase of exon model per million mapped reads): transcripts per thousand base transcripts per million mapped reads, summarizing length, expression, and number of genes, used to estimate sample expression; Num reads: estimated number of reads matched to each sample.
