# Supplementary material for: Differences in Gut Microbial Composition and Characteristics Among Three Populations of the Bamboo Pitviper (Viridovipera stejnegeri)
Source: Ecol Evol. 2024 Dec 17;14(12):e70742. doi: 10.1002/ece3.70742 (PMC11651729; doi:10.1002/ece3.70742)
Supplement: Supplementary file 2 — Appendix S2. Dominant bacteria of gut microbiota in different snake species. [file ECE3-14-e70742-s002.docx]

Appendix S2 Dominant bacteria of gut microbiota in different snake species

| **Status** | **Species** | **Sex** | **Location** | **Individuals** | **Collection** | **Dominant phyla** | **Dominant genus** | **References** |
| --- | --- | --- | --- | --- | --- | --- | --- | --- |
| Wild | *Ptyas dhumnades* | ♀ (pregnant) | Guilin, Guangxi, China | n = 3 | Feces | Bacteroidetes, Proteobacteria, Firmicutes | *Myroides, Bacteroides, Koukoulia, Citrobacter* | Li, Sun & Xu, 2021 |
|  |  |  | Xiangyang, Hubei, China | n = 3 |  |  | *Myroides, Bacteroides, Citrobacter, Cetobacterium* |  |
|  | *Crotalus horridus* | ♂ | Winona, Minnesota, USA | n = 1 | Stomach | Proteobacteria, Firmicutes, Bacteroidetes | */* | McLaughlin, Cochran & Dowd 2015 |
|  |  |  |  |  | Small intestine |  |  |  |
|  |  |  |  |  | Colon |  |  |  |
|  | *Rhabdophis subminiatus* | 2♀ (1Juvenile), 1♂ | Guangdong, China | n = 3 | Esophagus | Proteobacteria, Firmicutes, Bacteroidetes | *Fusobacterium, Mycoplasma, Bacteroides, Acinetobacter* | Tang *et al*., 2019 |
|  |  |  |  |  | Stomach |  |  |  |
|  |  |  |  |  | Large intestine |  |  |  |
|  |  |  |  |  | Small intestine |  |  |  |
|  |  |  |  |  | Cloaca |  |  |  |
|  | *Agkistrodon piscivorus* | / | Winston and Lafayette, Mississippi, USA | n = 3 | Large intestine | Bacteroidetes, Firmicutes, Proteobacteria | */* | Colston *et al*., 2015 |
|  |  |  |  | n = 3 | Cloaca | Proteobacteria, Firmicutes, Bacteroidetes |  |  |
|  |  |  |  | n = 8 | Small intestine |  |  |  |
|  | *Rhabdophis tigrinus* | / | Changyi District, Jilin, Jilin, China | n = 3 | Large intestine | Fusobacteria, Proteobacteria, Firmicutes | *Cetobacterium, Fusobacterium, Citrobacter, Bacteroides* | Tang *et al*., 2019 |
|  |  |  |  |  | Cloaca | Fusobacteria, Proteobacteria, Bacteroidetes | *Cetobacterium, Bacteroides, Fusobacterium, Citrobacter* |  |
|  |  |  |  |  | Small intestine |  | *Cetobacterium, Citrobacter, Fusobacterium, Bacteroides* |  |
| Farmed | *Elaphe carinata* | / |  | n = 3 | / | Firmicutes, Bacteroidetes, Proteobacteria | *Bacteroides, norank_f_Porphyromonadaceae, Enterococcus, Escherichia-Shigella* | Lu *et al*., 2019 |
|  | *Elaphe anomala* | / |  | n = 3 | / | Firmicutes, Bacteroidetes, Proteobacteria | *Bacteroides, norank_f_Porphyromonadaceae, Enterococcus, Clostridium_sensu_stricto_1* |  |
|  | *Elaphe schrenckii* | / |  | n = 3 | / | Firmicutes, Proteobacteria, Bacteroidetes | *Bacteroides, Bacillus, Enterococcus, Clostridium_sensu_stricto_1* |  |
|  | *Deinagkistrodon acutus* | / |  | n = 8 | Small and large intestine | Bacteroidetes, Firmicutes, Fusobacteria | *Bacteroides, Cetobacterium, Aeromonas, Providencia* | Qin *et al*., 2019 |
|  | *Naja atra* | / |  | n = 14 |  |  |  |  |
|  | *Ptyas mucosa* | / |  | n = 7 |  |  |  |  |
|  | *Elaphe carinata* | / | Xiangxi, Hunan, China | n = 6 | feces | Bacteroidetes, Proteobacteria, Firmicutes | *Bacteroides, Cetobacterium, Clostridium, Plesiomonas* | Zhang *et al*., 2019 |
|  | *Naja atra* | / | Yongzhou, Hunan, China | n = 6 |  |  |  |  |
|  | *Deinagkistrodon acutus* | / |  | n = 6 |  |  |  |  |
|  | *Ptyas mucosa* | / |  | n = 4 |  |  |  |  |
|  | *Python taeniura* | Juvenile |  | n = 32 | Large intestine, small intestine, and cecum | Firmicutes, Bacteroidetes, Proteobacteria | *Bacteroides, Rikenella, Lactobacillus, Synergistes* | Costello *et al*., 2010 |
|  | *Elaphe taeniura* | / | Yunnan, China | n = 5 | Jejunum, ileum, and rectum | Firmicutes, Bacteroidetes, Proteobacteria | *Enterococcus, Lactobacillus, Clostridium, Akkermansia* | Shi & Sun, 2017 |
